# Impact of ambient air pollution on gestational age is modified by season in Sydney, Australia

**DOI:** 10.1186/1476-069X-6-16

**Published:** 2007-06-07

**Authors:** Bin Jalaludin, Trish Mannes, Geoffrey Morgan, Doug Lincoln, Vicky Sheppeard, Stephen Corbett

**Affiliations:** 1 Centre for Research, Evidence Management and Surveillance, Sydney, Australia and School of Public Health and Community Medicine, University of New South Wales, Sydney, Australia; 2 NSW Public Health Officer Training Scheme, New South Wales Health Department, Sydney, Australia; 3 Northern Rivers Department of Rural Health, Sydney University, Lismore, Australia; 4 Queensland Institute of Medical Research, Brisbane, Australia; 5 Environmental Health Branch, New South Wales Health Department, Sydney, Australia; 6 Centre for Public Health, Sydney, Australia

## Abstract

**Background:**

The effect of individual pollutants and the period(s) during pregnancy when pollutant levels are likely to have most impact on preterm birth is not clear. We evaluated the effect of prenatal exposure to six common urban air pollutants in the Sydney metropolitan area on preterm birth.

**Methods:**

We obtained information on all births in metropolitan Sydney between January 1, 1998 and December 31, 2000. For each birth, exposure to each air pollutant was estimated for the first trimester, the three months preceding birth, the first month after the estimated date of conception and the month prior to delivery. Gestational age was analysed as a categorical variable in logistic regression models.

**Results:**

There were 123 840 singleton births in Sydney in 1998–2000 and 4.9% were preterm. Preterm birth was significantly associated with maternal age, maternal smoking, male infant, indigenous status and first pregnancy. Air pollutant levels in the month and three months preceding birth had no significant effect on preterm birth after adjusting for maternal and infant covariates. Ozone levels in the first trimester of pregnancy and spring months of conception and sulphur dioxide were associated with increased risks for preterm births. Nitrogen dioxide was associated with a decreased risk of preterm births.

**Conclusion:**

We found more protective than harmful associations between ambient air pollutants and preterm births with most associations non-significant. In view of these inconsistent associations, it is important to interpret the harmful effects with caution. If our results are confirmed by future studies then it will be imperative to reduce Sydney's already low air pollution levels even further.

## Background

Epidemiological studies addressing the relationship between ambient air pollution and birth outcomes are increasingly appearing in the scientific literature [1-4]. A recent systematic review of the impact of air pollution on pregnancy outcomes concluded that for preterm births the evidence as yet is insufficient to infer causality, but the available evidence justifies further studies [5]. The review suggests that the effect of individual pollutants and the period(s) during pregnancy when pollutant levels are likely to have most impact on preterm birth is not clear.

It is useful, then, to examine the impact of ambient air pollution and preterm birth in a variety of different sites to clarify the nature of the relationship. We examined this relationship in Sydney, Australia where ambient air pollution concentration is low compared with most cities where studies of the acute effects of air pollution have been conducted. For example, mean daily particulate concentrations in Sydney are lower than in any of the cities in the NMMAPS study in the USA [6].

In the present study we aimed to study the effect of prenatal exposure (in early and late pregnancy) to six common urban air pollutants [particulate matter less than 10 and 2.5 microns (PM10 and PM2.5 respectively), ozone (O3), nitrogen dioxide (NO2), carbon monoxide (CO) and sulphur dioxide (SO2)].

## Methods

Information on all births in metropolitan Sydney (total population approximately 3.8 million in 2001) between January 1, 1998 and December 31, 2000 was obtained from the Midwives Data Collection (MDC), New South Wales Department of Health. Metropolitan Sydney comprised the geographical areas as defined by the New South Wales Health Department. Three semi-rural local government areas were excluded as they were not adequately served by ambient air quality monitoring stations. The MDC is a population based surveillance system of all live births and stillbirths of at least 20 weeks gestation and at least 400 grams birth weight. Birth data include maternal demographic factors, pregnancy factors, details about the delivery and infant factors. Multiple births, babies born to women with gestational hypertension or gestational diabetes, and births greater than 42 weeks gestation were excluded from all analyses.

We obtained air pollution and meteorological data from the New South Wales Department of Environment and Conservation. The air pollution data included levels of: particulate matter (24-hour average; μg/m^3^), O3 (1-hour maximum; parts per billion [ppb]), NO2 (1-hour average; ppb), CO (8-hour average; parts per million [ppm]) and SO2 (1-hour maximum; ppb). Data were available for 14 monitoring stations within the Sydney metropolitan area. Monitoring stations were excluded if less than 80% of readings were available for the study period for each pollutant. Pollutant levels were analysed as continuous variables. We used SAS v8 statistical software (SAS Institute Inc., Cary, NC, USA) for all analyses.

For each birth, exposure to each air pollutant during gestation was estimated by calculating the mean concentration of each pollutant over the first trimester, the three months preceding birth, the first month after the estimated date of conception and the month prior to delivery. Air pollutant levels from monitoring stations were averaged to provide an estimate for the whole of metropolitan Sydney. Additionally, to provide a more sensitive estimate of exposure, the process was repeated matching air pollution data from each eligible monitoring station to births to mothers residing in postcodes within five kilometres of the monitoring station. Postcode areas were included in the five kilometres radius if more than 50% of the postcode was within the five kilometres radius.

Gestational age was analysed as a categorical variable (preterm birth: <37 weeks; term birth: ≥37 weeks but <42 weeks) in logistic regression models. Odds ratios (OR) and ninety-five percent confidence intervals (95%CI) are presented for a unit increase in air pollutant concentrations. We included in the regression models: sex of child; maternal age (in years); maternal smoking during pregnancy (Yes/No); gestational age at first antenatal visit (≤20 weeks or >20 weeks); whether mother identifies as being Aboriginal or Torres Strait Islander (indigenous status); whether first pregnancy; season of conception and socio-economic status (SES). Mean relative humidity and temperature at the time of pollutant exposure not significant in single variable models and were therefore excluded from all multivariate models. SES was measured using the Index of Relative Socio-economic Disadvantage (IRSD) of postcode of maternal residence. The Australian Bureau of Statistics constructs the IRSD on the basis of social and economic information collected in the population census, and assigns each postcode in NSW an IRSD value. The postcodes were then ranked and divided into quintiles. Note that autumn refers to March, April, May; winter refers to June, July, August; spring refers to September, October, November and summer refers to December, January and February. Potential covariates were initially examined in single variable logistic regression models. Significant covariates were then included in the basic model. Following the development of the basic model, air pollutant variables were then added to the basic model to determine associations between ambient air pollutants and preterm births. Two pollutant models were then constructed by adding a second air pollutant to the single pollutant models.

## Results

There were 123 840 singleton births of >20 weeks gestation in Sydney, in 1998–2000. 4.9% (6 011) of babies were born at less than 37 weeks gestation (Table 1). Mean gestational age was 39 weeks (standard deviation = 2 weeks). After adjusting for meteorological, maternal and infant factors, preterm birth was significantly associated with maternal age (OR 1.02, 95%CI 1.02–1.03), maternal smoking (OR = 1.62, 95%CI = 1.51–1.74), male sex of the child (OR = 1.22, 95%CI = 1.16–1.29), whether mother is an Aboriginal or Torres Strait Islander (OR = 1.60, 95%CI = 1.24–2.07) and first pregnancy (OR = 1.38, 95%CI = 1.30–1.45) (Table 1). SES exhibited a statistically significant association with preterm birth with an increasing risk of preterm births with decreasing SES. There was a significant reduction in the risk of preterm delivery if conception occurred in spring compared to summer (ORspring = 0.90, 95% CI = 0.83–0.97).

**Table 1:** Characteristics of all singleton births, Sydney, 1998 to 2000

	Preterm (<37 weeks) n = 6 011	Term (3742 weeks) n = 117 829	OR+ (95% CI)
Maternal age (years) Mean ± SD	30.3 ± 5.8	30.2 ± 5.2	1.02
Range	15.0–50.1	12.1–53.3	(1.02–1.03)
Maternal smoking during pregnancy Number (%)			
Yes	1 103 (18.4)	14 475 (12.3)	1.62 (1.51–1.74)
No	4 905 (81.6)	103 332 (87.7)	
Sex of infant Number (%)			
Male	3 369 (56.1)	60 415 (51.3)	1.22 (1.16–1.29)
Female	2 634 (43.9)	57 367 (48.7)	
Mother is Aboriginal or Torres Strait Islander Number (%)			
Yes	71 (1.2)	746 (0.6)	1.60 (1.24–2.07)
No	5 934 (98.8)	116 985 (99.4)	
Gestational age at first antenatal visit Number (%)			
>20 weeks	728 (12.5)	13473 (11.5)	1.05 (0.98–1.14)
≤20 weeks	5110 (87.5)	103 642 (88.5)	
Previous pregnancy Number (%)			
No	2897 (48.2)	50 060 (42.5)	1.38 (1.30–1.45)
Yes	3111 (51.8)	67 737 (57.5)	
IRSD Quintile Number (%)			
5* (Least disadvantaged)	1928 (32.1)	42 632 (36.2)	1.00
4	1363 (22.7)	25 761 (21.9)	1.18 (1.09–1.27)
3	1009 (16.8)	18 817 (16.0)	1.18 (1.09–1.28)
2	727 (12.1)	13 090 (11.1)	1.26 (1.15–1.38)
1 (Most disadvantaged)	984 (16.4)	17 529 (14.9)	1.27 (1.17–1.38)
Season of conception Number (%)			
Summer*	1540 (25.6)	28 764 (24.4)	1.00
Autumn	1513 (25.2)	29 453 (25.0)	0.97 (0.89–1.07)
Winter	1540 (25.6)	29 511 (25.1)	0.92 (0.79–1.07)
Spring	1418 (23.6)	30 101 (25.6)	0.86 (0.77–0.96)

*Referent group; +Adjusted for all other covariates in Table 1

Concentrations of CO, NO2, PM2.5 and SO2 were highest in winter and lowest in summer (Table 2). As expected there were high correlations between PM10 and PM2.5 (Table 3). Pollution levels varied between monitoring stations and correlation coefficients between monitoring stations ranged from 0.68 to 0.85 for CO, 0.46–0.85 for NO2, 0.53–0.94 for O3, from 0.67–0.91 for PM10, from 0.66–0.93 for PM2.5 and from 0.06–0.58 for SO2.

**Table 2:** Daily average air pollutant concentrations, Sydney, April 1997–December 2000

Air pollutant	All year	Summer	Autumn	Winter	Spring
PM10 (μg/m^3^) 24 hour average					
Mean (SD^1^)	16.3 (6.38)	18.2 (7.20)	17.0 (6.23)	14.5 (5.57)	15.7 (5.82)
Median (IQR^2^)	15.4 (7.51)	17.0 (8.13)	16.0 (7.92)	14.0 (7.47)	14.97 (6.95)
PM2.5 (μg/m^3^) 24 hour average					
Mean (SD)	9.0 (3.94)	8.7 (4.19)	9.4 (3.61)	9.5 (4.22)	8.5 (3.61)
Median (IQR)	8.2 (4.54)	7.7 (4.03)	8.9 (4.57)	8.8 (5.34)	7.6 (3.80)
O3 (ppb^3^) 1 hour maximum					
Mean (SD)	30.9 (14.19)	37.7 (18.89)	28.3 (12.54)	23.3 (5.78)	34.3 (11.87)
Median (IQR)	27.2 (13.43)	33.1 (26.20)	25.0 (12.51)	24.0 (5.56)	31.5 (11.05)
NO2 (ppb) 1 hour maximum					
Mean (SD)	23.4 (7.58)	18.0 (7.01)	25.1 (7.91)	25.9 (5.43)	24.4 (7.09)
Median (IQR)	23.0 (9.45)	16.0 (7.99)	23.8 (9.51)	25.7 (6.23)	23.2 (8.67
CO (ppm^4^) 8 hour maximum					
Mean (SD)	0.9 (0.68)	0.5 (0.24)	1.0 (0.69)	1.3 (0.84)	0.6 (0.35)
Median (IQR)	0.7 (0.73)	0.4 (0.32)	0.9 (0.82)	1.2 (1.07)	0.5 (0.49)
SO2 (ppb) 1 hour maximum					
Mean (SD)	3.6 (1.95)	3.6 (2.37)	3.6 (1.93)	3.8 (1.69)	3.6 (1.74)
Median (IQR)	3.3 (2.24)	3.1 (2.58)	3.3 (2.16)	3.6 (1.88)	3.2 (2.30)
Temperature (°C) 24 hour average					
Mean (SD)	17.7 (4.50)	22.4 (2.41)	18.3 (3.45)	12.4 (1.77)	17.7 (3.02)
Median (IQR)	17.8 (7.38)	22.4 (3.50)	18.4 (5.30)	12.1 (2.50)	17.5 (4.10)
Relative humidity (%) 24 hour average					
Mean (SD)	70.8 (11.49)	70.2 (10.26)	73.9 (10.45)	71.5 (12.06)	67.5 (12.13)
Median (IQR)	71.4 (14.99)	69.8 (13.33)	74.1 (12.82)	72.0 (15.97)	67.8 (15.43)

^1^SD = Standard deviation; ^2^IQR = Inter-quartile range; ^3^ppb = parts per billion; ^4^ppm = parts per million

**Table 3:** Correlation between air pollutants, Sydney, April 1997–December 2000

	PM_10_	PM_2.5_	O_3_	NO_2_	CO	SO_2_
PM_10 _(μg/m^3^) 24 hour average	1.00					
PM_2.5 _(μg/m^3^) 24 hour average	0.83^1^	1.00				
O_3 _(ppb^2^) 1 hour maximum	0.50	0.34	1.00			
NO_2 _(ppb) 1 hour maximum	0.48	0.65	0.25	1.00		
CO (ppm^3^) 8 hour	0.28	0.53	-0.21	0.60	1.00	
SO_2 _(ppb) 1 hour maximum	0.42	0.43	0.36	0.46	0.24	1.00

^1^p-value < 0.0001 for all correlation coefficients; ^2^ppb = parts per billion; ^3^ppm = parts per million

Table 4 presents associations between air pollutants and preterm births for all Sydney and for infants born to mothers living within 5 kilometres of air monitoring stations. All ORs have been adjusted for meteorological, maternal and infant covariates.

**Table 4:** Associations between air pollutants and preterm births, Sydney, 1998–2000

**Pollutant**	**Exposure period**							
	
	**One month preceding birth**		**Three months preceding birth**		**First month of gestation**		**First trimester**	
	
	Sydney OR‡ 95% CI	5 km OR 95% CI	Sydney OR 95% CI	5 km OR 95% CI	Sydney OR 95% CI	5 km OR 95% CI	Sydney OR 95% CI	5 km OR 95% CI
PM10 24 hour average	0.991 0.979–1.003	1.008 0.993–1.022	0.989 0.975–1.004	1.012 0.995–1.030	0.983** 0.973–0.993	0.957 0.914–1.002	0.987 0.973–1.001	1.009 0.978–1.041
PM2.5 24 hour average	0.984 0.962–1.008	1.042 0.997–1.089	0.981 0.952–1.011	1.111** 1.037–1.189	0.981 0.962–1.000	1.032 0.897–1.188	0.978 0.950–1.007	0.991 0.929–1.057
O3 1-hour maximum	0.998 0.987–1.006	0.984 0.906–1.069	0.998 0.989–1.007	1.011 0.910–1.124	1.004 0.995–1.012	1.604** 1.268–2.030	1.014** 1.005–1.022	0.807* 0.668–0.976
NO21 hour maximum	1.000 0.990–1.011	0.996 0.881–1.126	1.006 0.993–1.019	1.046 0.913–1.205	0.967** 0.958–0.975	0.589* 0.360–0.963	0.970** 0.959–0.980	1.052 0.741–1.493
CO 8 hour maximum	0.962 0.889–1.042	1.000 0.864–1.158	0.997 0.907–1.096	1.111 0.942–1.310	0.896** 0.842–0.953	1.030 0.685–1.548	0.774** 0.715–0.838	1.247 0.811–1.917
SO2 1 hour maximum	0.993 0.948–1.040	1.557* 1.017–2.382	0.977 0.898–1.063	2.330** 1.344–4.040	0.856** 0.819–0.894	1.378 0.876–2.167	0.834** 0.777–0.895	2.312** 1.290–4.145

‡OR estimates adjusted for: maternal age, maternal smoking during pregnancy, indigenous status, SES, gestational age at first antenatal visit, season of conception and parity. OR is for a one unit increase in air pollutant concentration; *p < 0.05; **p < 0.01

Air pollutant levels in the month and three months preceding birth had no statistically significant effect on preterm birth for all Sydney (Table 4). Most of the ORs were less than one.

Exposure in the first month of gestation decreased the risk of preterm birth for PM10 (OR = 0.98, 95%CI = 0.98–0.99), CO (OR = 0.90, 95%CI = 0.84–0.95), SO2 (OR = 0.86, 95%CI = 0.82–0.89) and NO2 (OR = 0.97, 95%CI = 0.96–0.98). There were no significant effects of O3 and a borderline significant effect of PM2.5 on preterm births.

The first trimester of pregnancy results were similar to those for exposure in the first month of pregnancy except that there was now a significant effect for O3. NO2 (OR = 0.97, 95%CI = 0.96–0.98), SO2 (OR = 0.83, 95%CI = 0.78–0.90) and CO (OR 0.77, 95%CI = 0.72–0.84) continued to demonstrate small but significant decreased risk of preterm births, and the O3 effect was now significant (OR = 1.01, 95%CI = 1.01–1.02). PM10 and PM2.5 levels had no effect on preterm birth. In two pollutant models, O3 levels in the first trimester of pregnancy remained a significant predictor of preterm birth (ORs between 1.02 and 1.08).

Eleven metropolitan air-monitoring stations had sufficient data for inclusion in the analysis for infants born within 5 kilometres of an air monitoring station. There were 65 814 eligible births in 1998–2000 and 3 596 (5.5%) were preterm.

Levels of all pollutants in the one month and three months preceding birth had no effect on preterm birth except for SO2 (OR = 1.56, 95%CI = 1.02–2.38 and OR = 2.33, 95%CI = 1.34–4.04 for one and three months respectively) and PM2.5 (OR = 1.11, 95%CI = 1.04–1.19 for 3 month only).

In the first month of pregnancy, O3 (OR = 1.60, 95%CI = 1.27–2.03) increased the risk of preterm birth whereas NO2 (OR = 0.59, 95%CI = 0.36–0.96) decreased the risk of preterm birth. In the first trimester of pregnancy, SO2 (OR = 2.31, 95%CI = 1.29–4.15) and O3 (OR = 0.81, 95%CI = 0.67–0.98) had significant effects on preterm births. CO, PM10 and PM2.5 had no effect on preterm birth in the first month or in the first trimester of pregnancy.

When comparing results (whether significant or not) for all Sydney with those for infants born to mothers living within 5 kilometres of air monitoring stations, the results are most consistent for exposure in the first month of gestation and for PM10 (both decreased risks), O3 (both increased risks) and NO2 (both decreased risk) (Table 4).

In two-pollutant models (data not shown), SO2 levels in the first and third trimester of pregnancy remained a significant predictor of preterm birth (ORs between 2.30 to 3.15), whereas O3 levels in the first three months of pregnancy did not remain a significant predictor of preterm birth.

Season of conception was a significant effect modifier in the association between all air pollutants and preterm birth in the first month and first trimester of pregnancy, and was present for all of Sydney and for infants born to women residing within 5 kilometres of air monitoring stations. The associations between air pollutant levels and preterm birth for the first trimester of pregnancy for all of Sydney by season of conception are presented in Table 5. Particulate matter demonstrated significantly increased risks for preterm birth if conception occurred in autumn and winter but a significantly decreased risk if conception occurred in summer. CO showed a decreased risk of preterm births across all four seasons whereas O3 only showed significant effects on preterm births if conception occurred in spring and summer. SO2 demonstrated significant effects on preterm births if conception occurred in autumn and winter and NO2 if conception occurred in winter.

**Table 5:** Associations between air pollutants and preterm births by season of conception^#^, Sydney, 1998–2000

	**Autumn n = 31 008**		**Winter n = 31 097**		**Spring n = 31 563**		**Summer n = 30 446**	
	
	OR^‡^	95% CI	OR	95% CI	OR	95% CI	OR	95% CI
PM_10 _24-hour average	1.462**	1.267–1.688	1.343**	1.190–1.516	1.119	0.973–1.288	0.913**	0.889–0.937
PM_2.5 _24-hour average	1.080	0.912–1.281	1.426**	1.264–1.608	1.156	0.972–1.375	0.879**	0.839–0.922
O_3 _1-hour maximum	1.012	0.987–1.037	0.989	0.972–1.007	1.093**	1.019–1.071	1.031**	1.023–1.040
NO_2_1 hour maximum	1.045	0.963–1.134	1.153**	1.066–1.247	1.002	0.933–1.077	0.957**	0.938–0.976
CO 8 hour maximum	0.114**	0.086–0.152	0.134**	0.062–0.281	0.584	0.283–1.462	0.633**	0.535–0.750
SO_2 _1-hour maximum	6.489**	4.365–9.648	1.323**	1.027–1.704	1.287	0.955–1.734	0.826**	0.759–0.898

^#^Analysis for the first trimester of pregnancy; ^‡^OR estimates adjusted for: maternal age, maternal smoking during pregnancy, indigenous status, SES, gestational age at first antenatal visit and parity. OR is for a one unit increase in air pollutant concentration; *p < 0.05; **p < 0.01

In two-pollutant models by season of conception (data not shown), the effect of PM10 on preterm birth for those infants conceived in autumn did not remain (non-significant ORs between 0.77 and 1.04). The effect of SO2 on preterm birth for those infants conceived in autumn remained significant when other pollutants were added to the model (ORs ranging from 6.48 to 9.25). The effect of PM2.5 on preterm birth for those infants conceived in winter did not remain (ORs between 0.97 and 1.03). The effect of O3 on preterm birth for those infants conceived in spring remained significant when other pollutants were added to the model (ORs between 1.07 and 1.10).

## Discussion

In this study, we examined the effects of ambient air pollution on preterm births across all of Sydney as well as for those infants born to women residing within 5 kilometres of air monitoring stations. We demonstrated mainly decreased risks in preterm births for exposure to NO2 and SO2 in the early months of pregnancy. There were few air pollution effects in the first month and in the three months preceding birth. Importantly, we also have shown that effects of air pollution on preterm births are modified by the season when conception occurred. For exposure to ambient air pollutants in the first trimester of pregnancy, significant adverse effects were generally observed in autumn and winter. However, there were also many associations that demonstrated a decreased risk of preterm births.

There are a number of strengths to our study. We were able to investigate the association between air pollutant concentrations and preterm births for a large number of births in metropolitan Sydney between 1998 and 2000 and we were able to test this association using information on infants born to women residing within 5 kilometres of an air monitoring station, again for a large number of births. We were also able to analyse the effect of all the common air pollutants (PM10, PM2.5, CO, NO2, SO2 and O3), as they are routinely monitored in metropolitan Sydney.

Importantly, in this study, we have been able to evaluate the effect of several important confounders, which have not been considered previously, including maternal age, maternal smoking during pregnancy, SES, gestational age at first antenatal visit and parity. Like pollutant levels, SES is measured at the ecological level and residual confounding by SES cannot be ruled out. Residual confounding by season may also be present. Vitamin D levels, which are related to sunlight exposure, and therefore season, are also related to gestational age [7], will vary within season and may be confounding the relationship between gestational age and pollutant exposure. We also cannot rule out confounding due to other risk factors for which we lacked data or were measured with error.

An important limitation of this study, as with most studies of ambient air pollution and health, is that of exposure misclassification. By using air pollutant concentrations averaged across Sydney, we assume that ambient pollutant concentrations represent an individuals' actual exposure to pollutants. This assumption does not account for time-activity patterns that may mediate exposure such as commuting habits, place or type of work or time spent outdoors. We attempted to overcome this by a sensitivity analysis of infants born to mothers residing within a 5 kilometres radius of air monitoring stations. However, even in smaller geographical areas there is the risk of misclassification because of variation in ambient air pollution levels due to topography, location of roadways and time-activity patterns.

Exposure misclassification in these situations would be expected to be non-differential and bias the point estimates towards the null. When ecological measures of exposure to air pollution, such as those obtained from fixed-site monitoring stations, are used as proxies for personal exposures, the estimates of pollutant effects are generally smaller than those based on exposure levels determined by personal sampling [8].

Pollutant levels (with the exception of SO2 for those births to mothers residing within 5 kilometres of air monitoring stations) in late pregnancy had no impact on preterm births for infants born in Sydney between 1998 and 2000. While other authors have observed effects of air pollutants on gestational age [3] and other birth outcomes [9-12], air pollutant levels in these regions often exceeded those observed in Sydney. Effects on gestational age were observed for CO, particulates and SO2 but an equal number of authors observed no effect of air pollutants in late pregnancy on birth outcomes [1,3,13].

The only other effects of pollutants on gestational age in this study occurred for pollutant levels in early pregnancy. We observed an increased risk of preterm birth for exposure to O3 in the first trimester of pregnancy for all of Sydney and for exposure to O3 and SO2 for infants born to women residing within five kilometres of an air monitoring station. Bobak [1] observed an effect of SO2 (OR = 1.27 for a 50 μg/m^3 ^increase in SO2) and total suspended particulates (OR = 1.18 for a 50 μg/m^3 ^increase in total suspended particulates) on gestational age in the first trimester of pregnancy in the Czech Republic. Ritz et al [3] observed an effect of PM10 levels (OR = 1.16 for a 50 μg/m^3 ^increase in PM10) on gestational age in Southern California. Xu et al [14] observed an effect of particulate and SO2 levels throughout pregnancy on gestational age in Beijing, China. Levels of particulate matter in those regions where an impact on gestational age was reported were higher than those recorded in Sydney in the study period. No other air pollutant appeared to affect gestational age in early pregnancy [1,9]. It is worth noting that, in our study, both NO2 and CO were associated with significantly decreased risks of preterm births.

In the main analyses, we found more significant protective effects than harmful effects of ambient air pollutants. The adverse effects were mainly for the five kilometre exposures and for SO2, whereas the protective effects were seen mainly for the Sydney-wide exposures and for NO2, CO and SO2. It does not appear that the spatial distribution of the air pollutants – with primary pollutants (NO2, CO and SO2) more likely to be localised and dependent on combustible sources and secondary pollutants (O3) more homogeneously distributed across Sydney – can explain these findings. These inconsistent results may be chance findings and need further investigation and corroboration.

We found that air pollutant effects varied according to season of conception and thus the time of year that the critical exposure period of early pregnancy occurs. Other investigators have observed and accounted for the impact of season on the relationship between pollutants and birth outcomes [9,14,15]. Wilhelm and Ritz [16] noted that the effect of residential proximity to heavy traffic roadways on preterm birth was greater for those whose third trimester of pregnancy fell in autumn/winter. In our study, for exposure to PM10 in the first trimester, infants conceived in autumn and winter had a 46% and 34% respectively of increased risk of preterm birth. PM2.5 levels had the most significant impact on those infants conceived in winter (OR = 1.42 for a 1 μg/m^3 ^increase in PM2.5). Xu et al [14] reported an increased estimate of effect of particulates on gestational age for births conceived in spring/summer. However, the higher levels of total suspended particulates reported by Xu et al [14] may partly account for the differing seasonal effects. We also conducted additional analyses for exposure in the first trimester and in the month prior to delivery with season of birth instead of season of conception. The odds ratios for all six air pollutants in the two set of models were similar. This may be due to the fact that both season of conception and season of birth could be adjusting for seasonal variation in a similar fashion.

In Sydney, CO exposure either decreased the risk or had no effect on preterm birth across all seasons whereas NO2 levels had the most significant impact on infants conceived in winter and O3 had the greatest impact on infants conceived in spring and summer (when O3 levels are highest). SO2 levels in early pregnancy had a large adverse impact on gestational age in those infants conceived in autumn and winter (OR 6.49 & 1.32 for a 1 ppb increase in SO2 respectively). Xu et al [14] also reported an increased estimate of effect of SO2 levels on gestational age (first trimester occurring in autumn/winter). Seasonal variation in reproductive outcomes has been well documented [17,18], although the reasons are not clear. Season is likely to be a proxy for other seasonal patterns of exposure, such as outdoor activity, which may in turn be related to reproductive outcomes. Vitamin D levels, which are related to sunlight exposure, and therefore season, are also related to gestational age [7]. It is also possible that an effect is detected in seasons where air pollutant levels are sufficiently high for an association to occur.

In two pollutant models, the Sydney-wide effect but not the five kilometre effect of O3 remained significant, and the five kilometre effect SO2 also continued to be significant suggesting independent effects of these two pollutants. However it should be noted that because of collinearity among air pollutants and that air pollutants may act as surrogates for other known or unknown air pollutants, the results from two pollutant models is often unclear and may not be meaningful. Further, these significant results were only a few of the many tests of significance that were conducted and we suggest that the results be viewed with caution.

Although air pollution has had little consideration as an important determinant of pregnancy outcomes, there is growing concern about its role as a reproductive health hazard. The biological mechanisms whereby air pollution causes preterm delivery remain to be determined. The hypothesised effect of air pollutants on reproductive health relate to a decreased in utero oxygen supply, resulting in a reduction of oxygen carrying capacity or indirectly through inflammation and changes in blood viscosity [2,19,20]. CO readily crosses the placenta to expose the foetus in utero, leading to rapid accumulation of carboxyhaemoglobin and reducing the oxygen carrying capacity of the blood [2] with subsequent adverse pregnancy outcomes including low birth weight and preterm birth [21]. In the first trimester, genetic mutations are generally considered the most important cause of placental abnormalities whereas complex vascular alterations are considered to be the main cause of placental abnormalities in the second and third trimesters [22]. The effect of air pollutant exposure during pregnancy on preterm birth has a plausible biological basis, however, the reported studies fail to show consistency in pollutants and periods during pregnancy where an effect occurs.

The lack of consistency in our findings may be due to the fact that air pollutants are correlated and that the common air pollutants may be surrogates for other measured or unmeasured air pollutants. In this study, when examining single pollutant models by season of conception, it appears that SO2 is an important pollutant, despite the fact that SO2 levels in Sydney are well below the national standard. In metropolitan Sydney, vehicular traffic is the primary source of SO2 and it is conceivable that SO2 is a marker for traffic related air pollutants in our study and therefore effects attributed to SO2 are due to traffic related air pollutants. Concentrations of pollutants measured at air monitoring sites in Sydney are typically below national air quality standards and generally lower than similar cities in developed countries, although seasonal conditions can cause the occasional exceedence of the national air quality standards [23].

We found more significant negative (protective effects) than positive (harmful effects) associations between air pollutants and preterm births. Given the number of comparisons made in this study, as occurs with most such studies, some (or many) of the significant findings, whether positive or negative, may well be spurious. In interpreting the literature on the effects of air pollutants on preterm births, and perhaps for all other health outcomes also, we need to examine for consistency and coherence in the overall results rather than focussing on individual significant results.

Should other research confirm the effects (whether positive or negative) of air pollutants on gestational age, it is possible that the effects observed for particular pollutants may be indicators of the urban air pollution mix. In this case the different air pollutants found to have a significant effect from city to city may be more related to the characteristics of the ambient air monitoring network than to any inherent differing effects of the monitored air pollutants.

## Conclusion

In conclusion, the results from our study offer additional information on the impact of ambient air pollutants on preterm births in Sydney, Australia and especially on the modifying effects of season of conception. In our many comparisons, we found more significant negative (protective effects) than positive (harmful effects) associations between air pollutants and preterm births with most associations being non-significant. In view of these inconsistent associations, both within our study and also when compared to other published studies, it is important to interpret the harmful effects with caution. It will be important to also corroborate our findings with future research to reach meaningful conclusions.

## Competing interests

The author(s) declare that they have no competing interests.

## Authors' contributions

BJ conceived the study and was involved in the statistical analyses and preparation of the manuscript. TM was involved in the statistical analyses and preparation of the manuscript. GM conceived the study and helped to draft the manuscript. VS, DL and SC assisted in the interpretation of the results and in critically revising the manuscript. All authors have read and approved the final manuscript.
